# Cocoa and Carob Supplementation, Alone or in Combination with Metformin, Protects against Hepatorenal Injury in Zucker Diabetic Fatty Rats

**DOI:** 10.3390/nu16183087

**Published:** 2024-09-13

**Authors:** Esther García-Díez, María Elvira López-Oliva, Jara Pérez-Jiménez, María Ángeles Martín, Sonia Ramos

**Affiliations:** 1 Instituto de Ciencia y Tecnología de Alimentos y Nutrición (ICTAN-CSIC), 28040 Madrid, Spain; esther.garciad@ictan.csic.es (E.G.-D.); jara.perez@ictan.csic.es (J.P.-J.); amartina@ictan.csic.es (M.Á.M.); 2 Sección Departamental de Fisiología, Facultad de Farmacia, Universidad Complutense de Madrid (UCM), 28040 Madrid, Spain; elopez@farm.ucm.es; 3 AFUSAN Group, Sanitary Research Institute of the San Carlos Clinical Hospital (IdISSC), 28040 Madrid, Spain; 4 CIBER de Diabetes y Enfermedades Metabólicas Asociadas (CIBERDEM), Instituto de Salud Carlos III (ISCIII), 28029 Madrid, Spain

**Keywords:** cocoa–carob blend, glucose homeostasis, insulin resistance, oxidative stress, apoptosis, liver and renal cortex, type 2 diabetic ZDF rats

## Abstract

The liver and kidneys are crucial for glucose homeostasis and are seriously damaged in diabetes. Cocoa and carob possess antidiabetic activity, but their hepatorenal protective effects, especially when combined with antidiabetic drugs, are unknown. The aim of this study is to investigate the effects of a cocoa–carob-supplemented diet (CC), either alone or in combination with metformin, on liver and kidney damage in Zucker diabetic fatty (ZDF) rats, a type 2 diabetes model. Male ZDF animals received a control or CC-supplemented diet, with or without metformin, and Zucker lean rats were fed the control diet. The CC-supplemented diet improved glucose tolerance and insulin resistance and alleviated functional and structural alterations in the diabetic liver and renal cortex. The CC-supplemented diet also ameliorated oxidative stress, downregulated apoptosis, and improved insulin signalling and glucose homeostasis. The combination of CC and metformin boosted several benefits as certain parameters related to morphological and structural alterations, apoptosis, oxidative stress, glucose homeostasis, and insulin resistance, were improved in comparison to animals receiving the CC-supplemented diet or metformin alone; these include the following: apoptotic index, Bax, hepatic insulin receptor or glutathione content, among others. These results demonstrate that the CC-supplemented diet alleviates the hepatorenal damage in type 2 diabetic ZDF rats, highlighting its potential alone or as an adjuvant therapy.

## 1. Introduction

Type 2 diabetes is a worldwide, highly prevalent disease caused by a metabolic disorder and characterised by sustained hyperglycaemia that leads to severe complications [1]. The liver and kidney are crucial organs that play an important role in maintaining systemic glucose homeostasis in the body. In diabetes, the liver and kidneys are severely damaged due to exacerbated oxidative stress, insulin resistance, and abnormal glucose homeostasis, which also contribute to maintaining hyperglycaemia in this disease [2,3,4]. Accordingly, an altered response of antioxidant defences, including glutathione (GSH) and the enzymes glutathione reductase (GR), glutathione peroxidase (GPx), and superoxide dismutase (SOD), has been reported in patients with diabetes [5,6]. Importantly, the transcription nuclear factor erythroid 2-related factor 2 (Nrf2), a key protein in the regulation of the antioxidant response, and several signalling pathways involved in the cellular response to the insult, such as sirtuin-1 (SIRT-1) and apoptosis-related proteins (caspases, Bcl-2 family proteins) are also dysregulated in diabetes [3,5]. In addition, insulin resistance leads to a situation in which the insulin receptor (IR) is less responsive to insulin, leading to the blockage of this pathway and altered glycogen synthesis in the diabetic liver and kidney [2,4,7]. Similarly, glucose uptake and gluconeogenesis in the liver and renal cortex are altered due to the dysregulation of key proteins involved in these processes, e.g., glucose transporter 2 (GLUT-2) and phosphoenolpyruvate carboxykinase (PEPCK) [2,4,7]. Consequently, therapies aimed at preventing oxidative stress, abnormal glucose homeostasis, insulin resistance, and the dysregulation of closely related processes, such as apoptosis, may represent an interesting approach against diabetes.

Cocoa is a rich source of phenolic compounds, mainly flavanols, and has demonstrated beneficial effects against diabetes and its associated complications [8,9]. Cocoa possesses antioxidant and antidiabetic properties that may contribute to improving glucose homeostasis, redox status, and insulin sensitivity in this disease [8,9]. Similarly, carob, a Mediterranean legume (*Ceratonia siliqua* L.), is rich in polymeric flavanols and other polyphenols, and has attracted attention for its potential health benefits, such as the maintenance of glucose homeostasis [10]. In addition, either the combination of antidiabetic drugs with phenolic compounds or the use of mixtures of extracts enriched with these natural compounds have recently been suggested as effective therapeutic interventions that could provide an efficient treatment [11,12]. In this context, metformin (M) is the first-line antihyperglycaemic drug used in patients with type 2 diabetes; however, the evaluation of the effect of dietary flavonoids or flavonoid-rich diets in diabetes alone or in combination with antidiabetic drugs, including metformin, has been scarcely studied. Moreover, the combination of drugs with these natural compounds may bring synergistic effects and more effective therapies against diabetes [13].

Taking all this into account, a polyphenol-supplemented food was prepared by combining cocoa powder and carob flour (60:40, CC). In our recent studies, this potential functional food has shown protective effects on pancreatic beta cells and the heart in diabetes [14,15]. In this context, it was investigated whether the potential antidiabetic preventive properties of this mixture extend to the associated liver and kidney damage in the animal model of type 2 diabetes. Thus, the aim of this study was to investigate the effects of a CC-supplemented diet, alone or in combination with metformin, on liver and kidney morphology, functionality, redox status, and the molecular mechanisms connected to the modulation of apoptosis and glucose homeostasis, namely insulin signalling (IR and glycogen content), gluconeogenesis (PEPCK), and glucose transporter levels (GLUT-2) in both tissues of ZDF rats. In addition, the potential enhanced benefits derived from the co-administration of the CC-supplemented diet and metformin were studied.

## 2. Materials and Methods

### 2.1. Chemicals and Materials

3,3′-diaminobenzidine (DAB), dichlorofluorescein (DCFH), dinitrophenylhidazine (DNPH), glucose assay kit, glutathione reductase (GR), reduced and oxidised glutathione (GSH and GSSG, respectively), hydrogen peroxide, nicotine adenine dinucleotide phosphate reduced salt (NADPH), o-phthaldehyde (OPT), proteinase K, *tert*-butylhydroperoxide (t-BOOH), and SOD kit were purchased from Sigma Chemicals (Madrid, Spain). Anti-IRβ and anti-phospho-IRβ (targeting phosphorylated Tyr1150/1151 of IR), anti-Bcl-x, anti-Bax, anti-SIRT-1, and anti-PEPCK antibodies were obtained from Santa Cruz (sc-711, sc-81500, sc-634, sc-526, sc-74465, and sc-32879, respectively; Qimigen, Madrid, Spain). Antiphospho-Nrf2 antibody (recognizing phosphorylated Ser40 of Nrf2) was purchased from Signalway antibody (Qimigen, Madrid, Spain). Additionally, α-tubulin and anti-β-actin were obtained from Abcam (Madrid, Spain) and Cell Signalling Technology (Werfen, Barcelona, Spain), respectively. The anti-GLUT-2 antibody was procured from Merck (Madrid, Spain). Kits for albumin and glycated haemoglobin (HbA1c) were acquired from Spinreact (BioAnalitica, Madrid, Spain) and the rat insulin ELISA kit was from Mercodia (AD Bioinstruments, Barcelona, Spain). The creatinine kit was purchased from Linear Chemicals S.L. (Barcelona, Spain), and alanine aminotransferase (ALT) and aspartate aminotransferase (AST) kits were obtained from BioSystems (Barcelona, Spain). Chemicals and materials for electrophoresis were acquired from BioRad Laboratories S.A. (Madrid, Spain).

### 2.2. Cocoa–Carob Diet

The cocoa–carob diet (CC) was produced by blending pure cocoa powder (generously provided by Idilia S.L., Barcelona, Spain) with carob flour (purchased from Casa Ruiz Granel Selecto S.L., Madrid, Spain) in a 60:40 ratio. This proportion was selected after performing a preliminary sensory analysis, which showed that 40% of carob was preferred by 60% of the participants [16]. A detailed description of this cocoa–carob product is given elsewhere [16].

Diets are based on the AIN-93G formulation, which supplies all the necessary nutrients for adult rats. The 10% cocoa–carob (CC) diet was prepared by incorporating 100 g/kg of the cocoa–carob blend in the AIN-93G formulation. Briefly, AIN-93G formulation without cellulose, starch, and sucrose was prepared by including these ingredients to adjust the carbohydrate and dietary fibre content based on the amount of the cocoa–carob blend added. The composition of these diets is presented in Table S1.

### 2.3. Animals and Diets

This study is an extension of a previous investigation in which animal descriptions and experimental designs were described in detail [14]. Briefly, male Zucker lean (ZL) animals and Zucker diabetic fatty [ZDF; ZDF/*crl-lepr* (fa/fa)] rats were obtained at 11 weeks of age from Charles River Laboratories (L’ Arbresle, France). Animals were housed in groups under controlled conditions, maintaining a temperature of 19–23 °C, humidity of 50–60%, and a 12 h light–dark cycle. Following a one-week acclimatization period, ZDF rats (n = 32) were randomly assigned to four groups: one group was fed a standard diet (ZDF), another group was administrated with metformin in the drinking water (300 mg/kg/day) and standard diet (ZDF-M), and the remaining two groups were fed the cocoa–carob-supplemented diet and received metformin (ZDF-M + CC) or not (ZDF-CC). Thus, the present study is focused on the effects of the combination of cocoa and carob, as well as the potential boosted effect of the CC supplementation in combination with metformin and does not allow to independently assess the responses derived from cocoa or carob, which have already demonstrated to be helpful against diabetes [8,9,10]. ZL rats were kept as a single group and received the standard diet. This healthy ZL group was included as an internal control of the diabetic situation in ZDF rats. Both diets were isocaloric, and all animals had *ad libitum* access to food and water. Daily food intake was recorded, and body weight was monitored weekly throughout the study.

At 24 weeks of age, animals were fasted overnight, and blood samples were taken for biochemical analysis. The serum was prepared by centrifugation of blood at 1000× *g* (4 °C, 10 min) and used for further biochemical determinations. Livers and kidneys were harvested, weighed, and divided into two portions: one was fixed in paraformaldehyde (PFA) for histological analysis, the other was frozen in liquid nitrogen and kept at −80 °C. All animal procedures were approved by the Regional Committee on animal care and use for experimental purposes (Comunidad de Madrid, Ref. number PROEX 079/19), and the animals were treated according to the European [17] and Spanish [18] legislation on the care and use of experimental animals.

### 2.4. Biochemical Analysis

Glycaemia was assessed weekly with an Accounted Glucose Analyser (LifeScan España, Madrid, Spain), and blood HbA1c was determined through latex turbidimetry according to the manufacturer’s instructions. Serum insulin, ALT, AST, and creatinine were assayed using commercial kits according to the manufacturer’s instructions. Insulin sensitivity was estimated by the homeostasis model assessment of insulin resistance (HOMA-IR) and the homeostasis model assessment of insulin sensitivity (HOMA-IS).

Urine samples were collected over a 24 h period by placing the animals in metabolic cages. Urinary glucose levels were determined using the glucose oxidase method, while albumin levels were assessed using a latex turbidimetry kit. Estimated glomerular filtration rate (eGFR, mL/min) was also calculated.

To measure hepatic and renal glycogen content, samples were boiled in 30% KOH, precipitated with 95% ethanol, and centrifuged at 840× *g* for 25 min. The glycogen was then resuspended, treated with H_2_SO_4_ and 5% phenol, and incubated for 30 min. The released glucose was measured at 530 nm.

### 2.5. Glucose Tolerance Test (GTT)

Overnight-fasted rats received an intraperitoneal injection of a 35% glucose solution (2 g/kg body weight), and blood samples were taken from the tail vein at baseline (t = 0) and at 15, 30, 60, 90, and 120 min following glucose administration. Blood glucose levels were measured using a glucometer (LifeScan España, Madrid, Spain). Overall changes in glucose levels during the GTT were assessed by calculating the area under the curve (AUC) above the baseline values.

### 2.6. Histological and Immunohistochemical Analysis

The liver and kidney were excised, fixed overnight in 4% PFA (dissolved in 0.1 M phosphate buffer pH 7.4), and then embedded in paraffin. Serial sections (4 µm) were mounted on glass slides, hydrated, and stained with Haematoxylin and Eosin (H&E), periodic acid–Schiff (PAS), or Masson’s trichrome according to the manufacturer’s instructions. To assess histological changes, 10 images of stained sections were acquired under a light microscope using a Leica DM LB2 microscope and a Leica DFC 320 digital camera (Leica, L´Hospitalet del Llobregat, Spain). Morphometric analysis was carried out with ImageJ 1.5.4 software (U. S. National Institutes of Health, Bethesda, MD, USA) and the colour deconvolution plugin. All slides were independently examined by two different researchers under blinded conditions. In the liver, the area occupied by lipid vacuoles in the H&E-stained sections from each rat was assessed across 10 fields and expressed as a percentage of the total surface area of these fields. Hepatic steatosis was graded according to the percentage of hepatocytes containing fat in accordance with the guidelines described by Kleiner et al. [19]. In the kidney, the glomerular and Bowman’s space were evaluated. Measurements were taken from the average of 10 cortical fields, or 10 glomeruli cut at the vascular pole for each kidney

Masson’s positive staining for fibrosis showing a blue colour to collagen and PAS-positive staining for glycogen or mucopolysaccharide deposits showing magenta-coloured granules in the cytoplasm of hepatocytes and renal tubules were determined using ImageJ 1.5.4 software (U.S. National Institutes of Health, Bethesda, MD, USA), and the colour deconvolution plugin was used to measure the stained area. The total area was determined and expressed as a percentage for statistical analysis.

Immunohistochemical analysis was performed on deparaffinised and rehydrated liver and kidney sections. After retrieving citrate antigen and quenching endogenous peroxidase, sections were incubated overnight at 4 °C with the primary antibodies to detect phospho-Ser (40)-Nrf2 and SIRT1.

The colour reaction was developed using a polymerised horseradish peroxidase-conjugated secondary antibody and counterstained with haematoxylin. Immunostaining intensity for each antibody was quantified using ImageJ 1.5.4 software (U.S. National Institutes of Health, Bethesda, MD, USA). Ten fields per section per rat were selected for analysis. Positive staining intensity was calculated as the percentage ratio of the stained area to the total field examined. 

The colour reaction was developed with a polymerised horseradish peroxidase-conjugated secondary antibody and counterstained with haematoxylin. The immunostaining intensity for each antibody was measured using ImageJ 1.5.4 software (U.S. National Institutes of Health, Bethesda, MD, USA). For analysis, 10 fields per section per rat were selected and evaluated. The positive staining intensity was calculated as a percentage of the stained area relative to the total field analysed.

### 2.7. Terminal Deoxynucleotidyl Transferase dUTP Nick End Labelling (TUNEL) Assay

In the liver and kidney sections, apoptotic cells were labelled *in situ* by detecting DNA fragmentation on paraffin-embedded sections using the TUNEL assay. After permeabilisation and rehydration, sections were incubated with proteinase K (20 μg/mL) for 15 min at 37 °C, followed by treatment with 3% hydrogen peroxide (5 min) to quench endogenous peroxidase activity. Then, slices were incubated with equilibration buffer for 10 min before immediately applying TdT-enzyme for 1 h at 37 °C. Sections were treated with peroxidase-conjugated streptavidin, stained with DAB, and counterstained with methyl green. The apoptotic index, representing the percentage of cells undergoing apoptosis, was calculated as the ratio of TUNEL-positive cells to the total number of cells counted across 10 consecutive fields using ImageJ 1.5.4 software (U.S. National Institutes of Health, Bethesda, MD, USA).

### 2.8. Fluorometric Analysis of Caspase-3 Activity

Activity of caspase-3 was analysed as described [20]. In brief, tissues were lysed and caspase-3 activity was assayed in a reaction mixture containing Ac-DEVD-AMC (N-acetyl-Asp-Glu-Val-Asp-7-amino-4-methylcoumarin) as substrate. To determine the enzymatic activity, fluorescence was measured at an excitation wavelength of 380 nm and an emission wavelength of 440 nm.

### 2.9. Preparation of Hepatic and Renal Lysates and Western Blot Analysis

Frozen liver and kidney samples were homogenised 1:5 (*w*:*V*) in extraction buffer [21,22]. Homogenates were then centrifuged at 14,000× *g* for 60 min. Supernatants were harvested, protein concentrations were measured using Bradford reagent, and samples were stored at −80 °C until use.

Equal amounts of protein were separated by SDS-PAGE. Then, membranes were incubated with the appropriate primary antibody, followed by treatment with peroxide-conjugated secondary antibodies: anti-rabbit (GE Healthcare, Madrid, Spain) or anti-mouse (Sigma, Madrid, Spain). Blots were developed using the ECL system (GE Healthcare, Madrid, Spain). Western blot normalisation was performed using β-actin or α-tubulin, and band quantification was carried out using a scanner and associated software.

### 2.10. Determination of ROS, Protein Carbonyl Groups, and GSH Content

ROS were quantified using an assay that detects the oxidation of DCFH to the fluorescent compound dichlorofluorescein (DCF) [21,22]. Briefly, homogenates of liver and renal cortex tissues were diluted 1:20 (*V*/*V*) with ice-cold Locke′s buffer and then incubated with 5 μM DCFH (30 min, 37 °C). Fluorescence was measured at an excitation wavelength of 485 nm and an emission wavelength of 530 nm.

Protein oxidation in liver and kidney cortex homogenates was quantified by analysing carbonyl group content, as previously described [21,22]. Absorbance was measured at 360 nm, and the carbonyl content was calculated as nmol/mg protein (extinction coefficient of 22,000 nmol/L/cm). Protein concentrations were analysed with the Bradford reagent.

GSH levels were measured using the Hissin and Hilf fluorimetric assay, as previously described [21,22]. Liver and renal cortex tissues were homogenised (1:20, *w*/*V*) in 50 mM phosphate buffer pH 7.0. Proteins were precipitated and subsequently centrifuged (10,000× *g*, 30 min). The assay was based on the reaction of GSH with OPT at pH 8.0. Fluorescence was measured at an excitation wavelength of 340 nm and an emission wavelength of 460 nm.

### 2.11. Analysis of GPx, GR, and SOD Activities

For analysis of antioxidant enzyme activity (GPx, GR, and SOD), frozen samples of liver and renal cortex were homogenised (1:5, *w*/*V*) in 0.25 M Tris, 0.2 M sucrose, and 5 mM DTT buffer pH 7.4 and centrifuged at 3000× *g* for 15 min. Determination of GPx activity relies on the oxidation of GSH by GPx, using t-BOOH as a substrate, which is coupled with the depletion of NADPH by GR [21,22]. GR activity was measured by monitoring the decrease in absorbance resulting from the oxidation of NADPH during the reduction of oxidised glutathione [21,22]. SOD activity was analysed using a commercial kit following the manufacturer’s instructions [21,22]. The assay relies on SOD’s ability to reduce superoxide anions, which is linked to the disappearance of Dojindo’s highly water-soluble tetrazolium salt (WST-1) to produce a dye. SOD activity was quantified by measuring the decrease in absorbance at 440 nm. Protein concentration was analysed using the Bradford reagent.

### 2.12. Statistics

Before conducting statistical analysis, data were assessed for homogeneity of variances using Levene’s test. For multiple comparisons, a one-way ANOVA was performed, followed by the Bonferroni test if variances were homogeneous or the Tamhane test if variances were not homogeneous. Statistical significance was determined at *p* < 0.05. Analyses were carried out using SPSS (IBM, Madrid, Spain) version 28.0.

## 3. Results

### 3.1. CC-Supplemented Diet Alone or in Combination with Metformin Improves Body Weight, Glucose Tolerance, and Insulin Sensitivity in ZDF Rats

The initial body weight was higher in all diabetic animals compared to the ZL rats, whereas the final body weight gain increased more in the ZL and ZDF-M rats than in all other diabetic animals, which showed similar values to those of the ZDF group (Table S2). The total food intake was lower in ZL rats than in all other ZDF groups; therefore, food efficiency was reduced in all diabetic animals (Table S2). All ZDF animals displayed greater liver and kidney weights than the ZL group, which exhibited the characteristic hepatomegaly and nephromegaly of diabetes (Table 1). This increase was also maintained for the liver-to-body weight ratio in all diabetic animals, whereas for the kidney/body weight ratio, only differences between the ZL and ZDF rats were observed (Table 1).

Glucose and HbA1c levels were reduced by the administration of CC, metformin, or both agents compared to the ZDF animals (Table 2). Interestingly, the combination of CC and metformin achieved levels comparable to the ZL rats for both parameters. Consistently, insulin resistance (HOMA-IR and HOMA-IS indices) increased in the ZDF rats vs. ZL, and all other experimental groups showed intermediate values for both indices compared to their lean and diabetic littermates (Table 2). Overall, these results suggest that the CC-supplemented diet alone or in combination with metformin alleviates glucose intolerance and insulin resistance in ZDF rats, with the combined treatment having a more remarkable effect on glucose control than either CC or the drug alone.

### 3.2. CC-Supplemented Diet, Metformin, and Their Combination Ameliorate Hepatic and Renal Dysfunction in ZDF Rats

To further explore the effects of the CC-supplemented diet, alone or in combination with metformin, on the liver and kidney of diabetic rats, hepatic ALT and AST, as well as serum and urinary parameters associated with the renal function, were assayed.

Hepatic ALT and AST levels were elevated in the ZDF rats compared to the ZL and diabetic animals receiving metformin alone or in combination with the CC-supplemented diet, whilst those fed with CC alone showed values intermediate to those of the ZL and ZDF rats (Table 2). Regarding renal function, serum creatinine increased in the ZDF animals compared to the ZL rats, whereas it decreased in the diabetic groups treated with metformin alone or in combination with the CC-supplemented diet (Table 2). Urine glucose decreased in metformin-treated animals compared to all diabetic groups and reached levels comparable to those of the ZL group when it was administrated in combination with the CC-supplemented diet (Table 2). Similarly, protein excretion decreased in all diabetic animals treated with CC and/or metformin compared to the ZDF rats, and the group receiving both agents showed the closest values to the lean littermates (Table 2). In addition, the increased eGFR in the ZDF rats was improved in the ZDF-CC group, and displayed values lower than those of the ZDF animals but higher than those of the ZL or metformin-treated diabetic rats (ZDF-M and ZDF-M + CC rats) (Table 2). All these changes induced by CC supplementation alone or in combination with metformin suggest that the CC-supplemented diet and the antidiabetic drug might contribute to alleviate hepatic and renal dysfunction in the ZDF rats.

### 3.3. CC-Supplemented Diet Alone or in Combination with Metformin Alleviates Hepatic and Renal Cortex Morphological Alterations in ZDF Rats

Diabetes is accompanied by clinical and morphological alterations in the liver and kidney, and to gain further insight into the potential protective effects of CC and/or metformin on structural changes, histological analyses were performed using H&E, PAS, and Masson’s trichrome staining in the liver and renal cortex sections.

**Figure 1:**
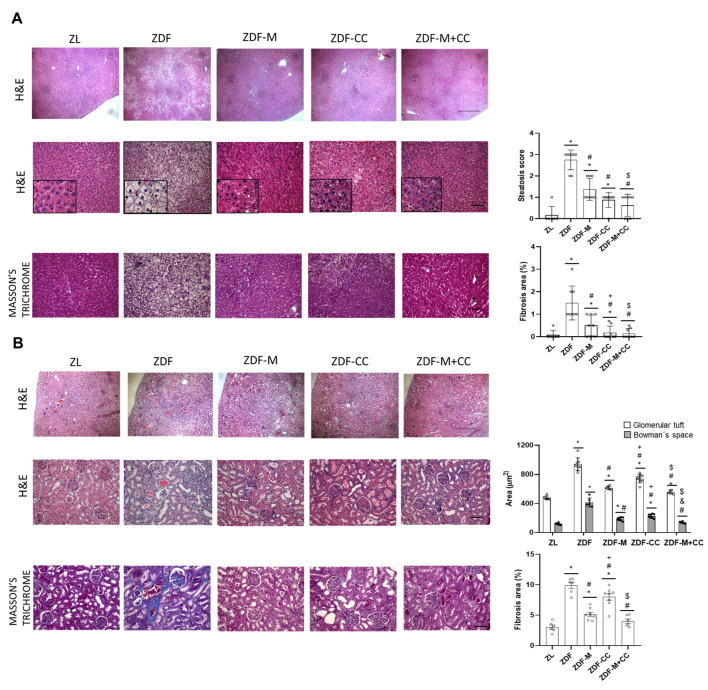
Effect of the cocoa–carob diet (CC), metformin (M), and their combination on the structure of the liver and renal cortex. (**A**) Representative hepatic tissue sections stained with H&E and Masson’s positive staining, and representations of the steatosis grade and percentage of fibrosis area. (Scale bar: 250 µm, magnification 4×; scale bar: 50 μm, magnification 20×; inserts 20 μm, magnification 40×.) (**B**) Representative renal cortical sections stained with H&E and Masson’s positive staining, and quantification of the glomerular tuft, Bowman’s space, and percentage of fibrosis areas. (Scale bar: 250 µm, magnification 4×; scale bar: 50 μm, magnification 20×.) Values are depicted as mean ± SD (n = 6–8). * *p* < 0.05 vs. ZL; # *p* < 0.05 vs. ZDF; + *p* < 0.05 ZDF-M vs. ZDF-CC; & *p* < 0.05 ZDF-M vs. ZDF-M + CC; $ *p* < 0.05 ZDF-CC vs. ZDF-M + CC.

The ZL rats displayed a normal hepatic architecture, characterised by typical hepatic lobules with a thin-walled central vein and hepatic cords extending toward the periphery, alternating with hepatic sinusoids (Figure 1A). The ZDF rats showed a signet-ring hepatocyte morphology due to extensive fat accumulation occupying between 34–66 % of the liver parenchyma (steatosis score = 2.7), which appeared as focal pericentral macro- and micro-vesicular fatty degeneration with the vacuolization of liver cells. In addition, the mild periportal interstitial mononuclear inflammatory infiltrates and enlargement of sinusoids affecting the centrilobular zone with a progressive loss of general organ structure were observed (Figure 1A). However, all other animal groups showed a notable restoration of normal hepatic architecture, with a decrease in the accumulation of fat droplets within the hepatocyte cytoplasm with diffuse macro-vesicular fat droplets (steatosis scores: ZDF-M = 0.9, ZDF-CC= 1.4, ZDF-CC + M= 0.6) and the regeneration of the hepatic parenchyma (Figure 1A).

As in the liver, the ZL rats showed a normal architecture of both renal corpuscles and tubules, whilst the ZDF animals exhibited an extensive pattern of renal disease characterised by glomerular, tubular, and renal interstitial injury. As illustrated in Figure 1B, the renal cortex of the ZDF rats showed glomerular hypertrophy and focal glomerulosclerosis, characterised by the regional adhesion of the enlarged glomerular tuft to Bowman’s capsule, associated with the expansion of the mesangial matrix and capillary filling and widening of Bowman’s space. Increased deposits of mucopolysaccharide and hyaline substances were also found in the cortex proximal tubules. In the ZDF rats, the renal medulla exhibited tubulointerstitial tissue with distended tubules covered by atrophic epithelial cells filled with protein casts and accompanied by increased inflammatory infiltrate around the tubules. The intrarenal arterial vessel showed a mild thickening of the walls and lipid droplets inside the capillaries and collagen deposition with hyalinisation. These alterations were largely prevented when the ZDF animals were supplemented with the CC diet and/or administered with metformin (Figure 1B).

In addition, although fibrosis was not very prominent in any animal, it was enhanced in the liver and kidney of the ZDF rats vs. ZL (Figure 1A,B). However, compared to the ZDF animals, rats receiving the CC diet and/or metformin showed ameliorated fibrotic damage (Figure 1A,B). Taken together, this may indicate that CC intake could contribute to minimise the adverse morphological hepatic and renal lesions in ZDF animals. Interestingly, the combination of diet and metformin treatment had a greater effect in preventing structural changes compared to the diet or drug alone.

### 3.4. CC-Supplemented Diet, Metformin, and Their Combination Prevent Apoptosis in the Liver and Renal Cortex of ZDF Rats

Diabetes could lead to apoptosis in the liver and kidney. Therefore, to gain insight into the molecular mechanisms of the protective effect of CC and/or metformin administration in the liver and renal cortex of the ZDF rats, apoptosis was analysed by TUNEL assay. Also, caspase-3 activity was measured, and key anti and pro-apoptotic Bcl-2 proteins were evaluated by Western blot.

**Figure 2:**
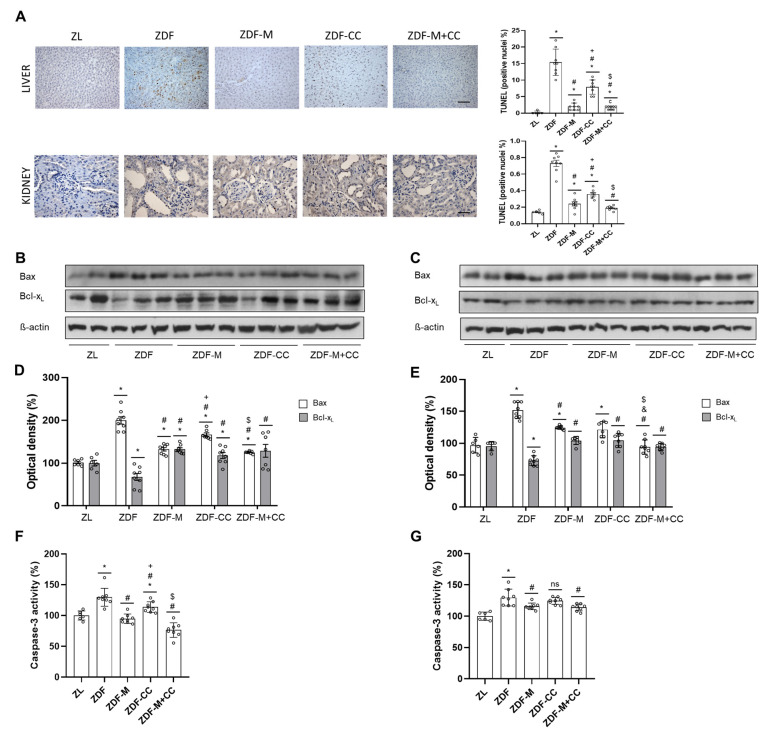
Effect of the cocoa–carob diet (CC), metformin (M), and their combination on apoptosis in the liver and renal cortex. (**A**) Representative TUNEL-positive hepatic and renal cells (scale bar: 20 μm, magnification 40×) and apoptotic index expressed as the percentage of positive nuclei relative to the total nuclei in the liver and renal cortex. Representative bands of Western blot analyses (**B**) in the liver and (**C**) in the renal cortex. Percentage levels of (**D**) hepatic and (**E**) renal cortex Bax and Bcl-x_L_ relative to the ZL animals (means ± SD, n = 6–8). Caspase-3 activity (**F**) in the liver and (**G**) renal cortex expressed as the percent of controls (ZL values). Values are shown as means ± SD of n = 6–8 animals. * *p* < 0.05 vs. ZL; # *p* < 0.05 vs. ZDF; + *p* < 0.05 ZDF-M vs. ZDF-CC; & *p* < 0.05 ZDF-M vs. ZDF-M + CC; $ *p* < 0.05 ZDF-CC vs. ZDF-M + CC; ns, not significant.

Histological sections showed a pro-apoptotic effect in the hepatocytes and renal cortical tubular cells of the ZDF animals, as assayed by TUNEL, whilst CC and/or metformin consumption remarkably prevented these changes (Figure 2A). TUNEL-positive cells were detected in the hepatocytes, and were scarcely observed in the glomeruli, with the majority of apoptotic cells localised in the renal cortical tubules (Figure 2A). In addition, the liver and renal cortex of the ZDF rats showed increased values of Bax with a concomitant decrease in Bcl-x_L_ levels (Figure 2B–E). However, in both tissues, supplementation with the CC diet and/or metformin administration reduced Bax values and raised Bcl-x_L_ levels (Figure 2B–E). Interestingly, Bcl-x_L_ levels were restored to control values (ZL animals) in the kidney of all treated ZDF rats and in the liver of the ZDF-CC + M group (Figure 2B–E). 

Similarly, caspase-3 activity was increased in the liver and renal cortex of the ZDF group; in both tissues, these values were comparable to those of the ZL rats when animals received metformin or the CC-supplemented diet or their combination, except for the liver in the ZDF-CC group, which showed levels intermediate to those of their lean and diabetic littermates (Figure 2F,G). These results suggest that the CC-supplemented diet and metformin may also contribute to hepatic and renal protective effects by preventing increased apoptosis in both tissues in diabetic animals.

### 3.5. CC-Supplemented Diet Alone or in Combination with Metformin Ameliorate the Hepatic and Renal Cortex Redox Imbalance in ZDF Rats

Oxidative stress is a major contributor to the induction of apoptosis in the liver and kidney in diabetes. To continue studying the protective effects of CC supplementation and metformin (alone or combined) in the liver and kidney of the ZDF rats, markers of oxidative stress (ROS and carbonyl groups), key antioxidant defences (GSH, GPx, GR, and SOD), and phosphorylated levels of Nrf2 and SIRT-1, which contribute to counteract oxidative stress and regulate the antioxidant response, were evaluated.

**Figure 3:**
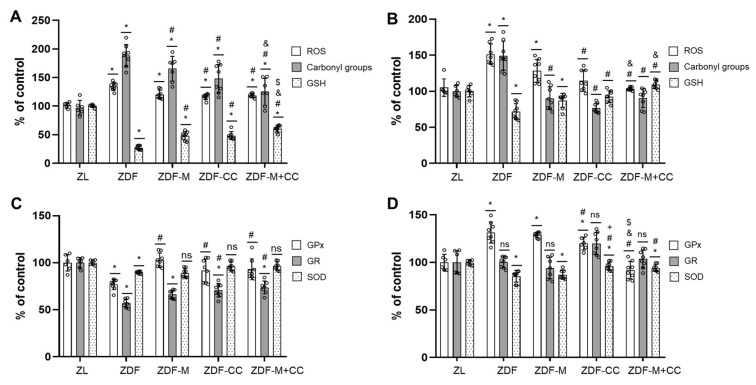
Effect of the cocoa–carob diet (CC), metformin (M), and their combination on the main antioxidant defences in the liver and renal cortex. Percentage of ROS levels, carbonyl groups, and GSH content (**A**) in the liver and (**B**) in the renal cortex relative to the ZL rats. Percentage of the activity of antioxidant enzymes GPx, GR, and SOD (**C**) in the liver and (**D**) in the renal cortex relative to the ZL animals. Data are shown as means ± SD of n = 6–8 animals. * *p* < 0.05 vs. ZL; # *p* < 0.05 vs. ZDF; + *p* < 0.05 ZDF-M vs. ZDF-CC; & *p* < 0.05 ZDF-M vs. ZDF-M + CC; $ *p* < 0.05 ZDF-CC vs. ZDF-M + CC; ns, not significant.

In the liver and renal cortex, ROS and protein carbonyl values were augmented in the ZDF rats when compared to all other experimental groups, whereas both parameters showed intermediate levels to those of the ZL and ZDF animals in the liver of all other diabetic rats (Figure 3A) and were similar to those of the ZL rats in the kidneys of both CC-fed groups (Figure 3B). In concert, GSH content was reduced in the ZDF animals compared to the ZL group in both tissues, but this effect was partially or completely suppressed in the liver and kidney of animals receiving the CC-supplemented diet (Figure 3A,B). Furthermore, in the liver, all enzymatic activities were decreased in the ZDF rats compared to the lean group, whereas GPx and SOD activities were restored to the ZL values in all other groups of animals, whereas GR activity was only partially recovered. Similarly, in the renal cortex, SOD activity was reduced in the ZDF vs. ZL rats, but its activity was partially improved with all the treatments, although both the CC-fed animal groups showed higher values than those receiving metformin alone (Figure 2D). Cortical renal GR activity was not modified in any group of rats, while GPx activity was enhanced in the ZDF animals compared to the ZL rats, being its activity partially or totally restored to control values (ZL animals) in ZDF-CC and ZDF-CC + M, respectively (Figure 3D).

**Figure 4:**
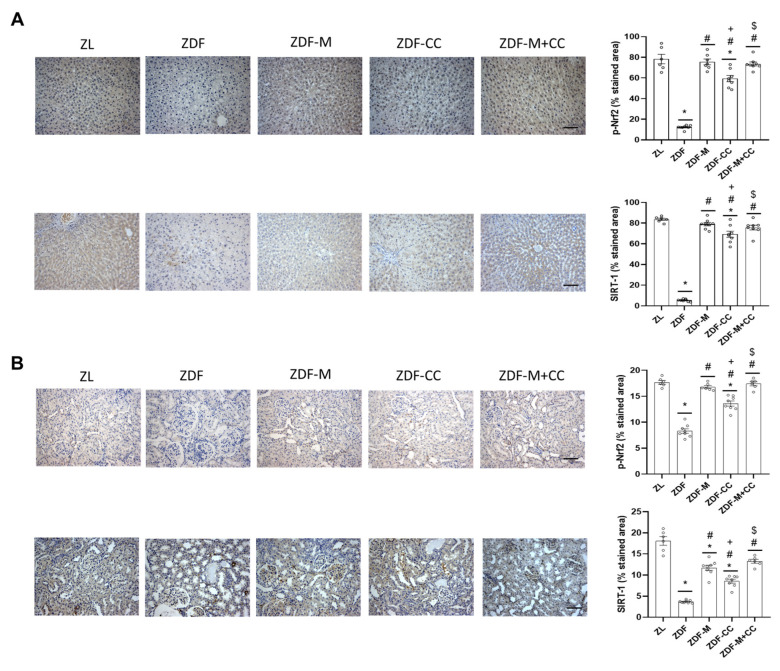
Effect of the cocoa–carob diet (CC), metformin (M), and their combination on levels of p-Nrf2 and SIRT-1 in the liver and renal cortex. Representative images of p-Nrf2 and SIRT-1 (scale bar: 50 μm, magnification 20×) and percentage of the stained area (**A**) in the liver and (**B**) in the renal cortex. Data are depicted as mean ± SD (n = 6–8). * *p* < 0.05 vs. ZL; # *p* < 0.05 vs. ZDF; + *p* < 0.05 ZDF-M vs. ZDF-CC; $ *p* < 0.05 ZDF-CC vs. ZDF-M + CC.

As shown in Figure 4, the liver and renal cortex of the ZDF rats showed the lowest levels of p-Nrf2 and SIRT-1 of all the animal groups, whereas rats fed with the CC diet alone exhibited intermediate levels of p-Nrf2 and SIRT-1 compared to their lean and diabetic littermates. Interestingly, as with most of the other parameters related to redox status in both tissues, the animals with values closer to or equal to those of the ZL group were rats receiving the CC-supplemented diet and metformin (Figure 3 and Figure 4).

Altogether, it is suggested that the CC-supplemented diet and metformin could contribute to restore the redox balance of the liver and renal cortex of the ZDF rats by modulating relevant antioxidant defences and closely related proteins, with the combined treatment being more effective than either the diet or drug alone.

### 3.6. CC-Supplemented Diet, Metformin, and Their Combination Improve Insulin Signalling in ZDF Rat Liver and Renal Cortex

The regulation of the insulin signalling pathway in the liver and kidney contributes to the maintenance of glucose homeostasis. In view of the results obtained, it was investigated whether supplementation with CC alone or in combination with metformin modulates the insulin route in the liver and renal cortex; thus, the phosphorylated and total levels of the first limiting step of this pathway, the levels of IR, and glycogen content were assayed by Western blot.

**Figure 5:**
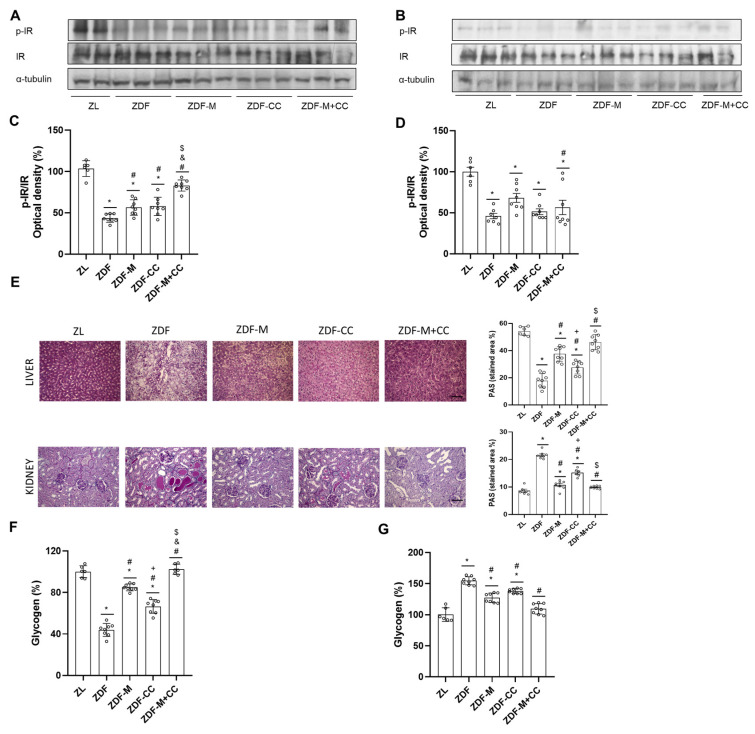
Effect of the cocoa–carob diet (CC), metformin (M), and their combination on insulin signalling in the liver and renal cortex. Bands of representative experiments for the phosphorylated and total levels of IR (**A**) in the liver and (**B**) in the renal cortex. Densitometric quantification of p-(Tyr)-IR and IR (**C**) in the liver and (**D**) in the renal cortex. Values (means ± SD) are expressed as a percentage relative to the ZL rats (n = 6–8). (**E**) Representative hepatic and renal cortex tissue sections stained with PAS (scale bar: 50 μm, magnification 20×) and the percentage of stained areas. Data are shown as mean ± SD (n = 6–8). Percentage of glycogen content in the (**F**) liver and (**G**) renal cortex. Values are expressed as a percentage relative to the ZL rats (means ± SD, n = 6–8). * *p* < 0.05 vs. ZL; # *p*< 0.05 vs. ZDF; + *p* < 0.05 ZDF-M vs. ZDF-CC; & *p* < 0.05 ZDF-M vs. ZDF-M + CC; $ *p* < 0.05 ZDF-CC vs. ZDF-M + CC.

Phosphorylated-(Tyr) IR levels are associated with an early response to insulin stimulation and were decreased in the ZDF rats compared to all other experimental groups in the liver and renal cortex (Figure 5A–D). In contrast, CC and/or metformin administration contribute to restore p-IR values in both tissues, with the ZDF-CC + M animals showing the highest levels or even similar levels to those of the ZL group in the renal cortex and liver, respectively (Figure 5A–D). Total insulin receptor (IR) levels were similar in all animal groups in both tissues (Figure 5A–D).

IR activation may contribute to the modulation of glycogen content. As shown in Figure 5E,F, hepatic glycogen levels decreased in the ZDF rats compared to all other animal groups, and its content was completely restored to ZL values in the ZDF-CC + M rats, in agreement with the increased p-(Tyr) IR levels. However, glycogen content was only partially restored in the livers of animals receiving metformin or CC alone (Figure 5E,F). On the contrary, an increase in the renal glycogen content was observed in the ZDF rats, whereas the ZDF-CC + M rats had values similar to those of the ZL group, and the ZDF-M and ZDF-CC animals showed values intermediate to those of their lean and diabetic littermates, as in the liver (Figure 5E,G).

Taken together, this suggests that in the liver and renal cortex, the CC-supplemented diet, and metformin prevent the blockade of the insulin route observed in the ZDF rats by regulating this pathway at the earliest step and contributing to the regulation of the glycogen content.

### 3.7. CC-Supplemented Diet Alone or in Combination with Metformin Regulates the Rate-Limiting Proteins of Gluconeogenesis and Transport of Glucose in the Liver and Renal Cortex of ZDF Rats

Increased gluconeogenesis and altered glucose uptake and reabsorption have been reported in the diabetic liver and renal cortex. To further investigate glucose homeostasis, key proteins involved in glucose production (PEPCK) and transport (GLUT-2) were analysed by Western blots in both tissues.

**Figure 6:**
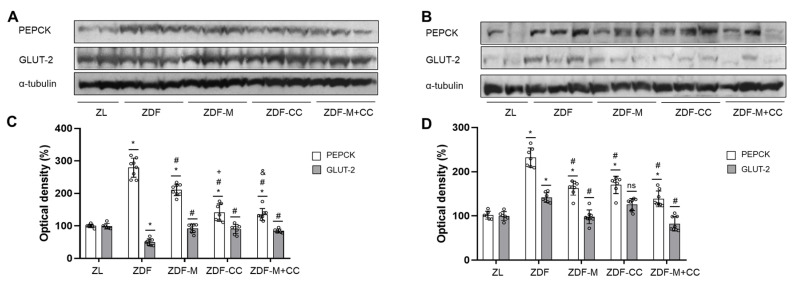
Effect of the cocoa–carob diet (CC), metformin (M), and their combination on levels of PEPCK and GLUT-2 in the liver and renal cortex. Bands of representative experiments (**A**) in the liver and (**B**) in the renal cortex. Densitometric quantification of PEPCK and GLUT-2 levels (**C**) in the liver and (**D**) in the renal cortex. Values (means ± SD) are expressed as a percentage relative to the ZL animal group (n = 6–8). * *p* < 0.05 vs. ZL; # *p* < 0.05 vs. ZDF; + *p* < 0.05 ZDF-M vs. ZDF-CC; & *p* < 0.05 ZDF-M vs. ZDF-M + CC; ns, not significant.

PEPCK levels augmented in the liver and renal cortex of the ZDF animals were compared to the lean group, and this effect was partially reversed in animals receiving the CC-supplemented diet and/or metformin (Figure 6). In addition, hepatic GLUT-2 values decreased in the ZDF animals compared to the ZL littermates, while rats fed the CC-supplemented diet and/or treated with metformin showed levels comparable to those of the ZL rats (Figure 6A,C). On the other hand, in the renal cortex, GLUT-2 levels were similar in the ZL and diabetic animals receiving the CC-supplemented diet and/or metformin, but the levels of this transporter were increased in the ZDF group (Figure 5A–C). All this suggests that the CC-supplemented diet alone or in combination with metformin may also help to preserve liver and kidney function and modulate glucose homeostasis in ZDF rats.

## 4. Discussion

Policies to prevent and treat diabetes and its associated complications are receiving increasing attention as morbidity and mortality rates continue to rise [1]. Different antidiabetic drugs are available, but their side effects and the occurrence of drug resistance are a concern. Flavonoids have been shown to have protective effects against diabetes due to their antioxidant and antidiabetic properties, among other beneficial activities [8,9]. In addition, a growing body of literature points out the synergistic effects of combined flavonoid administration [23,24]. However, there is a lack of evidence on the benefits of combining these natural compounds, especially with antidiabetic drugs, in the diabetic milieu in key organs such as the liver and kidneys. The present study demonstrates, for the first time in a well-established model of type 2 diabetes, ZDF rats, the hepatorenal protective effect of a mixture of cocoa and carob (CC) and a more promising effect in combination with metformin, the first-line drug used in diabetes.

In diabetes, suppressed body weight gain has been related to an increased energy loss [25], as shown in this study. However, the absolute liver and kidney weights, and the ratio of these organ weights to the body weight increased in the diabetic animals (hepato- and nephromegaly), whereas these parameters decreased when the animals received CC and/or metformin (Table 1). These results agree with previous reports showing an improvement in the diabetic condition by reducing weight loss and decreasing hepatic and renal weights in animals receiving polyphenol-rich extracts [24,25].

Diabetes also causes changes in the structure and function of the liver and kidneys [12,26,27] and importantly, at present, it is widely accepted that the alterations in one of these organs impair the function of the other [2]. In this regard, reduced renal function has been associated with the progression of liver fibrosis in diabetic patients [28]. Thus, in the present study, all the recoveries in histological changes could be related to the improvement in hyperglycaemia and could be associated with the attenuation of weight loss, reduced hepato- and nephromegaly, as well as with the alleviation of renal hyperfiltration, which in turn is connected with reduced levels of serum creatinine and urinary glucose and albumin [26,27]. In fact, improved glucose homeostasis delayed the progression of diabetes and its complications, including nephropathy [2,29], which is in line with studies supporting that various hepatic and renal alterations can be reversed at a certain point by good glycaemic control [27,30,31]. Overall, the results obtained here are consistent with previous studies showing that extracts rich in flavonoids or pure phenolic compounds, as well as the drug metformin alone, have protective effects against diabetes-induced functional and structural alterations in the liver and kidneys of diabetic rodents [25,26,32,33]. However, it is worth noting that none of these studies evaluated the effects of combining a mixture of polyphenol-rich foods with any antidiabetic drug, including metformin, on the liver and kidneys in type 2 diabetes, and most of them did not study both organs.

Chronic hyperglycaemia-mediated apoptosis in the liver and kidney contributes to the dysfunction of both organs in diabetes [29,34]. However, various pure polyphenols and flavonoid-rich extracts, as well as metformin, have been shown to attenuate apoptosis cell death in the liver and kidney, thus contributing to the alleviation of the diabetic milieu [21,26,27], in agreement with the present results.

Oxidative stress induced by chronic hyperglycaemia is closely related to apoptosis and is an important pathophysiological mechanism underlying the structural and functional changes in the liver and kidney in diabetes [5,29,34]. In fact, hyperglycaemia increases glucose oxidation and the release of large amounts of ROS, leading to cellular oxidative damage that can anticipate to liver and kidney injury [26]. In this study, the administration of CC and/or M to the diabetic animal groups enhanced several markers of oxidative stress, i.e., ROS generation, the oxidation of proteins, and altered antioxidant defences. In addition, the levels of redox-sensitive proteins Nrf2 and SIRT-1 were enhanced by CC supplementation and/or M administration in comparison to the ZDF rats. Consistently, several studies have demonstrated the ability of flavonoid-rich extracts and diets, as well as pure phenolic compounds, alone or combined with each other or with metformin, to prevent hepatic and renal oxidative stress by activating antioxidant defences via Nrf2 or SIRT-1, raising the antioxidant capacity against oxidative insult and contributing to the maintenance of redox balance [21,22,23,35], as shown here. Moreover, it is worth noting that the reduction in hyperglycaemia mediated-oxidative stress could also contribute to the amelioration of fibrosis and apoptosis in both organs [12,25], which is in line with the present study. Interestingly, the exploration of oxidative stress-related molecular pathways, such as endoplasmic reticulum stress, whichplays a major role on main altered biological processes in diabetes, is challenging and deserves further investigation.

Alterations in insulin signalling, gluconeogenesis, and glucose uptake in key organs critically involved in glucose homeostasis, such as the liver and kidneys, occur in diabetes [4]. Thus, chronic hyperglycaemia impairs hepatic and cortical renal insulin signalling at the early steps, i.e., IR recruitment and activation are inhibited [4,29], as shown in the current study. However, in agreement with the present results, it has been reported that the administration of a cocoa-rich diet, metformin, and flavonoid-rich extracts from mulberry leaves or basil ameliorated insulin resistance by increasing the total or tyrosine-phosphorylated levels of IR in the liver or renal cortex of diabetic rodents [26,30,33]. Importantly, insulin resistance has been associated with liver fibrosis and albuminuria in diabetic patients [36,37]. Similarly, the administration of CC and/or metformin improved insulin sensitivity and alleviated albuminuria and fibrosis in the liver and kidneys of the ZDF rats.

Activation of insulin signalling can lead to increased glycogen synthesis for energy storage and the suppression of glucose output by inhibiting PEPCK [2,4,29]. Thus, restoration of hepatic glycogen content has previously been shown in the liver of diabetic animals receiving metformin, flavonoid-rich foods, or mixtures of polyphenols [23,31,38], as presented here. Interestingly, the administration of flavanol-rich foods prevented glycogen accumulation in the renal tubules of diabetic rats, which is in agreement with the current study [30,32]. This differential regulation of the glycogen content in the liver and renal cortex of diabetic animals has been associated with the progression of injury induced by the disease [39]. In addition, it should be highlighted that the glycogen content in the renal cortex, as assessed here, has been scarcely studied, and that increased levels of hepatic glycogen have been reported in rats in the pre-diabetic period [31], which decrease in the later stages of the disease [38]. Taken together, CC supplementation alone or with metformin could improve both hepatic and renal insulin resistance and contribute to glucose homeostasis by modulating the glycogen content of both tissues in ZDF rats.

Another mechanism to regulate glucose homeostasis is the modulation of glucose production. Only the liver and renal cortex are capable of gluconeogenesis, and PEPCK plays a crucial role in this process as a rate-limiting enzyme [2,3]. In this line, in diabetes, low levels of this key enzyme in the liver and renal cortex could contribute to the inhibition of gluconeogenesis in both tissues, thus reducing hyperglycaemia in diabetic rats, which is in line with the present results [24,30,31]. Furthermore, it has been suggested that reduced levels of IR in hepatocytes and renal proximal tubules contribute to the maintenance of high glucose levels through enhanced gluconeogenesis [4,40]. Therefore, the current results suggest that CC and/or metformin may also contribute to a reduction in hyperglycaemia by regulating gluconeogenic PEPCK enzyme levels in the ZDF rats, with insulin signalling playing a role in this process.

Glucose uptake also contributes to the maintenance of glucose homeostasis [2,4]. Indeed, impaired glucose uptake in the liver and peripheral tissues leads to hyperglycaemia in diabetic patients [41]. Thus, reduced hepatic levels of GLUT-2 have been demonstrated in ZDF rats [42], as shown here, but the transporter levels were restored to the control values (ZL group) in ZDF animals receiving CC and/or metformin. Consistently, pure phenolic compounds, meat enriched with carob fruit extract, and antidiabetic drugs, including metformin, increased hepatic GLUT-2 levels [11,43], suggesting the normalisation of post-receptor insulin signalling and the restoration of hepatic insulin sensitivity. Similarly, the kidney also plays a relevant role in glucose homeostasis [29], although glucose transporter modulation in this organ has been disregarded. In the current study, upregulated levels of GLUT-2 were detected in the renal cortex of the ZDF animals, as previously shown [42]. This alteration contributes to the aggravation of hyperglycaemia and insulin resistance and has been associated with the accumulation of glycogen in the renal tubules [32], which is in agreement with the present results. Interestingly, a cocoa-rich diet and phlorizin supported the restoration of renal GLUT2 levels in diabetic rodents, demonstrating a renoprotective effect [30,44], as shown here. Therefore, the improved glucose homeostasis and insulin sensitivity in diabetic animals could also be related to the modulatory effect of CC and/or metformin on the hepatic and renal GLUT-2 content.

## 5. Conclusions

This study demonstrates that a cocoa–carob-rich diet alleviates hepatorenal dysfunction in ZDF diabetic rats by preventing structural alterations, including fibrosis, ultimately improving glucose homeostasis. These benefits were related to the alleviation of insulin resistance and to the modulation of the glucose transporter GLUT-2 and PEPCK gluconeogenic enzyme levels. In addition, the CC-rich diet promoted redox balance through the restoration of antioxidant defences, thereby mitigating oxidative stress and apoptosis. Importantly, several of these benefits were further improved by the combination of a CC-rich diet and metformin compared to either agent alone, demonstrating the efficacy of combining natural bioactive compounds with antidiabetic drugs in the treatment of diabetes, and also highlighting the potential of the CC-rich diet against hepatorenal complications alone or as an adjuvant therapy in the diabetic milieu.

## Figures and Tables

**Table 1:** Liver and kidney weights of rats fed standard (ZL and ZDF) and cocoa–carob-rich (ZDF-CC) diets, and metformin-treated rats fed standard (ZDF-M) and cocoa–carob-rich diets (ZDF-CC + M) ^†^.

	ZL	ZDF	ZDF-M	ZDF-CC	ZDF-CC + M
Liver weight (g) Liver-to-body weight ratio (×100) Kidney weight (g) Kidney-to-body weight ratio (×100)	10.15 ± 1.14 * 2.69 ± 0.25 * 1.36 ± 0.14 * 0.36 ± 0.03 *	18.71 ± 1.49 * 4.77 ± 0.42 * 1.78 ± 0.28 * 0.47 ± 0.09 *	19.68 ± 2.04 * 4.50 ± 0.67 * 1.60 ± 0.22 * 0.38 ± 0.07 ^ns^	18.12 ± 1.93 * 4.55 ± 0.60 * 1.70 ± 0.23 * 0.44 ± 0.05 ^ns^	14.89 ± 1.25 * 3.63 ± 0.27 *^#&$^ 1.47 ± 0.23 ^ns^ 0.38 ± 0.03 ^ns^

^†^ Data represent the means ± SD. * *p* < 0.05 vs. ZL; # *p* < 0.05 vs. ZDF; & *p* < 0.05 ZDF-M vs. ZDF-M + CC; $ *p* < 0.05 ZDF-CC vs. ZDF-M + CC; ns, not significant.

**Table 2:** Biochemical parameters connected with the metabolism and liver and kidney function in rats fed the standard diet (ZL and ZDF) and treated with metformin (ZDF-M) and fed with the cocoa–carob-rich (ZDF-CC) diet and treated with metformin (ZDF-CC + M) ^†^.

	ZL	ZDF	ZDF-M	ZDF-CC	ZDF-CC + M
**Serum** Glucose (mmol/L) Insulin (µU/mL) HbA1c (%) AUC glucose (mM/min) HOMA-IR HOMA-IS ALT (U/L) AST (U/L) Creatinine (mg/dL)	5.34 ± 0.63 * 11.26 ± 0.73 * 4.32 ± 0.21 * 1010 ± 118 * 1.98 ± 0.17 * 365.50 ± 31.83 * 71.00 ± 5.81 * 240.00 ± 10.58 * 0.20 ± 0.01 *	16.22 ± 1.19 * 10.56 ± 0.51 ^ns^ 20.28 ± 1.71 * 3025 ± 227 * 8.98 ± 0.82 * 81.65 ± 7.80 * 257.38 ± 19.56 * 695.51 ± 38.27 * 0.33 ± 0.01 *	8.58 ± 0.76 *^#^ 12.68 ± 0.63 ^ns^ 6.24 ± 0.23 ^#^ 1804 ± 419 *^#^ 4.06 ± 0.36 *^#^ 157.55± 8.42 *^#^ 77.66 ± 8.84 ^#^ 228.57 ± 22.86 ^#^ 0.28 ± 0.02 *^#^	9.65 ± 1.28 *^#^ 14.53 ± 1.81 ^ns^ 15.75 ± 0.93 ^*#+^ 1758 ± 557 *^#^ 5.35 ± 0.57 *^#^ 138.17 ± 13.55 *^#^ 213.45 ± 10.83 *^#+^ 607.35 ± 28.36 *^#+^ 0.33 ± 0.03 *^+^	6.42 ± 0.60 ^#&$^ 13.81 ± 0.47 ^ns^ 3.58 ± 0.23 ^#$^ 1181 ± 472 *^#^ 4.45 ± 0.40 *^#^ 160.24 ± 15.16 *^#^ 71.50 ± 8.97 ^#$^ 241.50 ± 12.84 ^#$^ 0.30 ± 0.02 *
**Urine** Glucose (mmol/24 h) Albumin (mg/24 h) eGFR (mL/min)	1.78 ± 0.20 * 4.13 ± 0.25 * 0.48 ± 0.08 *	147.42 ± 9.26 * 72.34 ± 7.13 * 1.25 ± 0.11 *	85.94 ± 16.30 *^#^ 42.60 ± 4.69 *^#^ 0.66 ± 0.10 ^#^	136.37 ± 11.51 *^+^ 38.64 ± 6.69 *^#^ 0.99 ± 0.10 *^#+^	1.87 ± 0.32 ^#&$^ 17.76 ± 3.67 *^#&$^ 0.45 ± 0.04 ^#$^

^†^ Data represent the means ± SD. * *p* < 0.05 vs. ZL; # *p* < 0.05 vs. ZDF; + *p* < 0.05 ZDF-M vs. ZDF-CC; & *p* < 0.05 ZDF-M vs. ZDF-M + CC; $ *p* < 0.05 ZDF-CC vs. ZDF-M + CC; ns, not significant.

## Data Availability

Data are available upon request to the authors. The data are not publicly available due to principle of confidentiality.

## Supplementary Materials

The following supporting information can be downloaded at: https://www.mdpi.com/article/10.3390/nu16183087/s1. Supplementary Table S1: composition of the experimental control (C) and cocoa–carob-rich (CC) diets (g/kg dry weight). Supplementary Table S2: body weight data, food intake, and food efficiency of rats fed with standard (ZL and ZDF) and cocoa–carob-rich (ZDF-CC) diets, and treated with metformin and fed with standard (ZDF-M) and cocoa–carob-rich diets (ZDF-CC + M).
