# Supplementary material for: Cocoa and Carob Supplementation, Alone or in Combination with Metformin, Protects against Hepatorenal Injury in Zucker Diabetic Fatty Rats
Source: Nutrients. 2024 Sep 13;16(18):3087. doi: 10.3390/nu16183087 (PMC11434748; doi:10.3390/nu16183087)
Supplement: Supplementary file 1 [file nutrients-16-03087-s001.zip › nutrients-3175208-Supplementary Tables.pdf]

Supplementary Table S1. Composition of the experimental control (C) and cocoa-carob-rich (CC) diets (g/Kg dry weight).

	C	CC
Casein	140	140
Dextrose	155	155
Sucrose	100	92
Soybean oil	40	40
t-BHQ[a]	0.008	0.008
Mineral mix.	35	35
Vitamin mix.	10	10
L-Cystine	1.8	1.8
Choline bitartrate	2.5	2.5
Cellulose	100	44.5
Corn starch	415.7	379
Cocoa-carob powder	-	100

[a]*tert*-butylhydroquinone

Supplementary Table S2. Body weight data, food intake and food efficiency of rats fed with standard (ZL and ZDF) and cocoa carob-rich (ZDF-CC) diets, and treated with metformin and fed with standard (ZDF-M) and cocoa-carob-rich diet (ZDF-CC+M)*.

	ZL	ZDF	ZDF-M	ZDF-CC	ZDF-CC+M
Initial body weight (g)	281.33 ± 8.40	343.11 ± 10.90*	339.13 ± 9.53*	338.13 ± 15.52*	342.75 ± 8.05*
Final body weight (g)	377.67 ± 13.90	394.63 ± 6.49[ns]	434.00 ± 30.01*	399.43 ± 15.69[ns]	420.71 ± 19.23[ns]
Body weight gain (g in 12 weeks)	99.33 ± 9.52	55.00 ± 5.02*	93.33 ± 19.49[ns]	64.26 ± 5.32*	78.14 ± 13.74*#&$
Total food Intake (g in 12 weeks)	1561.93 ± 55.78	2491.11 ± 37.82*	2379.93 ± 36.41*	2475.25 ± 31.53*	2382.25 ± 42.50*
Food efficiency	6.56 ± 0.34[a]	2.12 ± 0.21*	4.29 ± 0.20*#	2.53 ± 0.21*+	3.26 ± 0.46*#$

* P< 0.05 vs. ZL; # P< 0.05 vs. ZDF; + P< 0.05 ZDF-M vs. ZDF-CC; & P< 0.05 ZDF-M vs. ZDF-M+CC; $ P< 0.05 ZDF-CC vs. ZDF-M+CC; ns not significant. ZL: Zucker lean; ZDF: Zucker diabetic rats; ZDF-M: Zucker diabetic rats treated with metformin; ZDF-CC: Zucker diabetic rats fed with a CC-rich diet; ZDF-CC+M: Zucker diabetic rats treated with metformin and fed with a CC-rich diet.
